# Microscopic Anatomy or Histology of Man

**Published:** 1853-10

**Authors:** 


### REVIEW DEPARTMENT.

### ARTICLE XIII.

*Mikroskopische Anatomie oder Gewebelehre des Menchen, von Dr. A. Koelliker, Professor der Anatomie und Physiologie, in Wuertzburg. Leipsig,* 1852. *Von den Zaehnen.* § 143 *to* § 155.

*Microscopic Anatomy or Histology of Man. By Dr. A. Koelliker, Professor of Anatomy and Physiology, in Wuertzburg. Leipsig,* 1852. *Upon the the Teeth.* § 143 *to* § 155. By Christopher Johnston, M. D. Member of the Medico-Chirurgical Faculty of Maryland. Member of the American Medical Society in Paris. Lecturer in the Maryland Medical Institute.

It is curious at this day to observe, that while men of most nations are eagerly seeking to abridge the toil necessarily attendant upon faithful observation, so that the most opposite opinions are maintained with warmth, and yet give but assertions as a result, the German labors unceasingly and unobtrusively, assures himself that he sees before he calls upon others to believe, weighs well his interpretation of facts, and allows himself to say "I do not know" when his experience is inadequate to enable him to wrest a coveted truth from nature. There are, to be sure, exceptional cases, but the favorable instances, we are confident, are sufficiently familiar to obviate the necessity, on our part, of adducing any other than the author, whose book is the subject of this notice.

Under the head of "organs concerned in digestion" we find the teeth, considered in their anatomical relations and intimate constitution—"the soft parts of the teeth, the alveolar periosteum, the tooth germ, and the gums" are next described—then follow a history of development, general applications and deductions accompanied with historical references, and a brief summary of the nature and condition of the organs in the lower animals; the pathological relations are then shown; and the article is terminated with directions for the complete examination and study of the teeth.

"Dentine," says our author, page 56, "consists of a fundamental substance and many small tubes, *canaliculi dentium*, which course in it. The first is, in fresh teeth, even in the thinnest sections, perfectly homogeneous, without trace of composition out of cells, fibres or other elements. After the extraction of the salts of lime of the dentine it manifests on the contrary, a strong tendency to tear parallel with the tubuli into coarse fibres, from which still finer fibrils of 0.002 or 0.003 line in breadth may then be separated, which, however, immediately declare themselves as artificial products by their irregular form; and, indeed, their existence is wholly dependent upon the fact that the tubuli, lying close together, proceed parallel to each other throughout the dentine."

And again, page 60, * * "but I have not been able to convince myself of their existence as a distinct element of dentine. The cartilage of the tooth appears, indeed, to be most delicately fibrous, but this appearance, is produced in certain conditions only of the tubuli that course in it, and the rough, granulated, fibroid masses, which, however, can never be isolated in considerable length, are too far apart to present regular, constant relations."

We shall perceive, when in another place we advert to the author's modes of manipulation, that he has not arrived at the above conclusion without careful deliberation; nevertheless, Henle speaks of these "fibres" as a "fasciculus of microscopic fibrils, being analogous in color to those of the middle coat of the arteries, and in form to the external fibres of the crystalline lens. They are slightly flattened, pale, granular, rough, almost jagged, especially upon the lateral edges, by which they touch each other: their width reaches 0.0029 fr. line. * * The separation of the dental substance into fibres, is not artificial, as might be supposed—it cannot be doubted, that the formation is primitive. The history of the development of the tissue, will furnish us a decisive proof of the fact."*

*** Henle, Anatomie générale—Truduction Française, Paris, 1843, p. 431.**

Page 60. "Neither is there a cellular structure of the fundamental substance, as Nasmyth assumes, although, indeed, in soft tooth-cartilage, it displays here and there between the canaliculi an indistinct cross marking that reminds us somewhat of enamel."

We will presently have occasion to call up the question of "cellular structure," and, therefore, pass over it briefly for the moment. Concerning the "cross markings," we are not disposed to coincide with the author, having satisfied ourselves of the occasional existence of striæ in simple sections of the teeth of young people, and most conspicuously in carious teeth at points but little remote from the seat of disease; nevertheless, a delusive appearance of "indistinct cross markings," is sometimes presented, and depends upon the aberration of light caused by tubuli beyond the focus, which, when closely examined, (with 500 diameters,) are found to contain air bubbles, arranged in *serial lines.*—See also, Valentin, loc. cit. page 731. But thin laminæ prepared from teeth of every sort, which had been gently treated with acid, invariably displayed the cross lines so plainly as to enable us to recognize them as the boundaries of intertubular compartments. A more prolonged action of the acid, completely eradicates all trace of these divisions; until finally, there is left nothing but the "*baccated fibres,*" of Nasmyth, which, in truth, are *canals* constricted at regular intervals.

The author is more fortunate in *isolating* the canaliculi, not in fragments only, but in their whole length. Schwann, Henle, Nasmyth, Müller, and Todd and Bowman, had previously succeeded in obtaining partial views of tubuli freed from the fundamental substance; and Henle, after giving their dimensions, affirms that the "walls are immeasurably thin."* And Valentin,† has also assured himself of their individuality; for he describes, as a result of his own observations, intercanalicular, cuneiform

* Henle, loc. cit.

† G. Valentin. Wagner's Handwoerterbuch der Physiologie. 1 Band, 1842, page 729.

fibrils, insoluble in acetic acid. Koelliker treats sections of teeth with muriatic or nitric acid, until the intertubular substance shall have disappeared, whereupon the tubuli, and even some of the fine ramifications, remain perfectly distinct.

He says, page 61: "The tubular structure is always manifest in the larger ones from the inner part of the dentine when the reagents have acted well, and there is plainly seen a clear cavity and a tolerably thick wall, which will often escape observation in the others, (the narrower,) and these also display the manifold bendings, and occasional thickening and thinning, which are easily explained by their softness and viscosity. Nasmyth's '*baccated fibres*,' are nothing else than these. * * * Thus much, at least, may be inferred from this observation, that the substance which immediately surrounds the canaliculi, is thicker and more resistent than the rest, and may hence, with propriety, be regarded as the proper wall."

The figure, page 62, gives, assuredly, no idea of the "*fibres*" in question.

Not a little interesting is the description we find given of what the author terms the "*interglobular spaces*" in the dentine, already mentioned by Owen* in their simplest condition as vestiges of the "dentinal cells," well described as to their outline, by Tomes,† and more recently by Hassall,‡ who found in them "a resemblance to oil globules." The author differs from Tomes as regards the contents of the spaces, and from both Tomes and Owen as concerns the molecular agency of their formation.

P. 65. The interglobular spaces "are irregular cavities of the dentine, limited by spheroidal protuberances, being never entirely absent in any tooth, and are found more particularly in the crown and in the neighborhood of the cement. In the crown they appear most frequently near the enamel, * * * and likewise further inwards; always, however, in lines which correspond to the contour-lines. * * * The projections, which have precisely the same appearance as the dentine, and

*R. Owen, Odontography, &c. London, 1840,-1845—p. 464.

†J. Tomes—a Course of Lectures on Dental Physiology and Surgery. London, 1848—p. 44.

‡A. H. Hassall—The Microscopic Anatomy of the Human Body. Amer. Ed., N. York, 1851—plate xxxvii, fig. 5.

are also perforated by canals, I term dentine spheroids. * * * The signification of the interglobular spaces and dentine-spheroids will be clearly set forth under the head of development of the teeth, but I will here remark, that their presence is normal in teeth in the course of formation, but in fully grown teeth, on the other hand, it is a defect of first formation. * * The spaces contain, during life, no fluid, but a soft substance with tubuli, exactly like the dentine, coinciding with the tooth-cartilage, and which, after long maceration in muriatic acid, is evidently more resistant than the fundamental substance of the fully calcified tooth, and consequently may be isolated precisely as the canaliculi."

Our author accounts for the transverse markings of the enamel prisms in the same manner as Tomes,* and avoids all mention of their "doubtful nature" as adduced by Todd and Bowman,† or of the "set of prismatic cells," of Carpenter,‡ which, after treatment of a tooth in acid, remain as "moulds in which the mineral substance was deposited." However, he observes, that "when the acid has acted, a viscous frame-work remains, in which, oftentimes, one *thinks* he distinctly sees a tubulus." As for the interprismatic canals of writers, which should convey a nutrient fluid from the dentine, he adds: "assuming this, is going farther than direct observation will justify;" and that, too, without denying such canals to exist. P. 103.

At page 76 we find an account of an "enamel investing membrane, * * which supplants the coronal layer of cement in the herbivora, &c., not in man alone, but also in the quadrumana and the land carnivora, and which I am constrained to regard as different from the cement," (p. 116;) and farther, (p. 110,) the identity of the "investing membrane" and the cement-layer of animals is denied on the ground that "they are chemically and morphologically different—moreover, in certain animals, the former is superadded to the latter."

P. 76. "The *enamel investing membrane*, of a thickness of 0.0004–0.0008, is intimately connected with the enamel, but

* J. Tomes, loc. cit., p. 164.

† The Physiological Anatomy and Physiology of Man; R. B. Todd and W. Bowman. (Reprint,) Philadelphia, 1850, p. 531.

‡ Principles of Human Physiology, by W. B. Carpenter, London, 1846, p. 155.

does not, however, pass over the cement. It is found in teeth at every age, unless worn away by use or otherwise destroyed, and by moistening a tooth-section with concentrated sulphuric acid is demonstrable in fragments. The pellicle separates itself more slowly in dilute acid, so that its connection with the whole tooth and its entire expansion may be examined. The membrane is a calcified, structureless expansion, * * which, mostly from adherent impurities, appears granular and of a yellowish hue, but otherwise regarded, gives no certain indication of farther composition. The only one to be perceived in it is a minute granulation, and upon the side next to the enamel a more or less indistinct representation in the form of small, polygonal facets, which oftentimes are little pits for the reception of the ends of the enamel prisms. What chiefly distinguishes this organic substratum of the envelop is *its great resistance to chemical agents*, whereby the membrane becomes an admirable protection to the crown of the tooth; it also undergoes no change by maceration in water, and is not dissolved by boiling. As little is the enamel-investing membrane affected even at a high temperature, by acetic, muriatic, sulphuric and nitric acids, only it assumes a yellowish tint in the last named. In the alkaline carbonates and in caustic ammonia it remains unaltered. Treated with caustic potassa and soda, it becomes white and somewhat flocculent, but its continuity endures."

We have thus given at length the foregoing account of a constituent of the tooth which hitherto has not received merited attention in virtue of its eminently protective character, and consequently of its practical importance. We would not, however, wish to be understood as announcing a discovery, for already Ficinius* and Klenke† had given their observations to the world, attributing to the pellicle in question a fibrous structure, which opinion Koelliker very justly rejects as erroneous.

We simply refer to the *system of canals* connecting the "granular layer" of Tomes with the cement, of which the partial observations of Tomes‡ and others had previously gained for them a knowledge of the essential§ characters—and also to the cavi-

* *Ficinius*, üeber das Ausfallen der Zaehne und das Wesen der Zahucaries—*Journal für Chirurgie* von *Walther und Ammon*, 1846.

† *Klenke*—die Verderbniss der Zaehne—Leipsig, 1850.

‡ Tomes, loc. cit. p. 47, &c.

§ Well seen in Hassell, loc. cit. plate xxxvi, fig. 7.

ties or "*spaces*" resembling those found in the enamel, which our author upon frail grounds supposes to be entirely the result of resorption. We pause, however, to cite a passage, replete with good sense, which corroborates the testimony of Valentin,* concerning the difficulties attendant upon the study of the peripheral nerve-endings:

P. 85. "With regard to the terminations (of the nerves) I would wish to express myself in favor of the looped form, yet I confess that the thing is still involved in doubt so long as the primitive fibrils of the possible loops shall not have been followed from twig to twig, the which has hitherto not been seen by any one."

But, while we admire the candor of the learned professor, we must suppose him unacquainted with the researches, &c., (posthumous) of Nasmyth, A. D, 1849, wherein the looped form of termination is distinctly affirmed, and illustrated by a drawing.

Sections 150, 151, treat of the development of the teeth, and accord with the sterling contribution of *Goodsir*, upon the same subject. Indeed, we find it frankly stated, that, although the author has examined, (in this connection,) p. 94, "human teeth only, he has not seen all that Goodsir has met with, yet has observed the most essential matters precisely as that writer has done." Again he says, (p. 93,) Goodsir "is the first who has placed the formation of the teeth in a proper light. I am thus certainly of opinion, that in all essential particulars, he has most correctly seen, nevertheless, his observations, even though almost universally accepted, have been confirmed by *no one*, if we except some slight accordances of Todd and Bowman, and Nasmyth; and they are called in question by Marcusen. (*Marcusen*, ueber die Entwickelung der Zæhne der Sæugethiere. St. Petersburg, 1850.)"

We cannot forbear noticing the error which the author has inadvertently committed, that "Goodsir's researches have not been confirmed by others," for we find Todd and Bowman† distinctly to affirm that they have in most particulars had an opportunity of verifying the excellent observations of Mr. Goodsir;

*G. Valentin, Encyclopēdie Anatomique. Nēvrologie (Trad. Française,) p. 28. Paris, 1843.

† Todd and Bowman, loc. cit. p. 532.

and Henle* not only refers to Goodsir's detailed description of the first phases of the development of the teeth, "which prove that Arnold has correctly observed," denying at the same time, however, his description to be strictly exact—but reproduces his views at length; and if farther testimony were wanting, we might adduce the author's own statement that as *far as he has gone* he perfectly coincides with Goodsir. As for Marcusen, his investigations cannot invalidate those of the last named writer, because "they are confined to the lower mammifera; and besides avoiding all mention of the sacculi, he speaks of the dental groove as a *lusus naturæ.*"

At p. 98, we find the announcement very much at variance with our accepted views, that

"*The præformative membrane* is without any signification as to tooth-formation; and it does not deserve its name, which, however, may be permitted to it on account of its brevity;" and (p. 101) a denial of the existence of the "basement membrane" or "structureless membrane," substituting for it a substratum of the reticular areolar tissue somewhat thinner than 0.008 line, and leaving to the *membrana adamantinæ* nothing but a row of cylindrical cells, "a true cylinder epithelium." A a discussion of these points would be unprofitable, we therefore pass on to consider the author's general views of calcification and ossification.

We had wished to have seen some definite idea attached to these terms, which the present condition of physiological and chemical science would justify. It is true, that the word *transformation* (umwandlung) serves to render the generally accepted opinion; but we are made acquainted by the beautifiul researches of Robin,† Lebert‡ and Broca,§ amongst others, that the embryonic cells of *animals* occupy this relation, that all the elements of *products* result directly from their *metamorphosis*, while the elements of *constituents* are formed by *substitution* of

* Henle, loc. cit. p. 438.

† *C. Robin.* Du microscope et des Injections. Paris, 1849. Comptes rendus et Mēmoires de la Sociētē de Biologie. Paris, 1850.

‡ *H. Lebert.* Maladies Scrofuleuses et Tuberculeuses. Paris, 1849.

§ *Broca.* Rapport ā Sociētē Anatomique. Paris, 1851.

these elements in place of the original transitory embryonic cells. "It might be said," observes Robin,* "that there is transformation of cartilage into bone if in this case the cartilagine, a substance which yields chondrine, simply incrusted itself with a calcareous phosphate, so as still to furnish chondrine by boiling bones. It might be said, that there is transformation of one tissue into another, if the first should come to possess, little by little, other anatomical elements or to present another texture, preserving, nevertheless, the same immediate principles, especially the organic substances which are proper to it. But from the moment that *in the place* occupied by a tissue one discovers not only other anatomical elements disposed differently than at first, but besides these, elements formed of other immediate principles, evidently it should not be stated that there has been *metamorphosis*, but rather *substitution of anatomical elements for others*, and consequently of one tissue for another."

Our own observations would lead us to offer an additional objection to the ancient theory of ossification besides the mechanical impossibility of deposit of "calcareous salts," in "cartilage" or "blastema" without a proportionate, an obvious augmentation of volume of the recipient tissue, and this is, that in an "ossifying cartilage," the particles substituted for those of the transitory nidus, are not in the condition of "salts" but of elementary osseous molecules. Of this, Valentin† was, in part sensible, since from experiments upon bones treated with acids, and by calcination, he derives the conviction, that a great part of the earthy salts are in a state of *chemical combination* with the bone-cartilage, but adds, that an equally considerable portion of them, is mechanically contained in the lacunæ and canaliculi. These "chemical and mechanical deposits," however, appear to him insufficient to account for all the phenomena of ossification, whereupon, a new element is introduced, namely, "an organic metamorphosis in the bone-cartilage itself."

* *Robin and Verdeil.* Chemie Anatomique et Physiologique. Paris, 1853. T. 3, p. 367.

† G. Valentin—Wagner's Handwoerterbuch der Physiologie, 1 Band. p. 724.

In fine, the modes of ossification are simply two: 1, by "*substitution,*" and 2, by "*invasion,*" the latter differing from the former in this particular, that, as in the cranial bones, for instance, the cartilaginous tissue is occupied or "invaded" in proportion as it extends itself little by little into other tissues, while "substitution" follows the creation of the cartilaginous web at an appreciable interval. The essential nature of these two modes, is not different.

We have then in the *fœtus*, a dense *fundamental substance*, which, with its *cavities*, and their contained "*corpuscles*," or masses of organic particles that in the child are replaced by the *cells of cartilage*, constitutes the temporary cartilaginous tissue. In the *fundamental substance*, there occurs a deposit of osseous molecules, supplanting the molecules of that substance, and surrounding the *cavities*, at first opaque, but becoming afterwards more homogeneous as the "substitution" is more complete. The cavities are now encroached upon, the corpuscles or cells disappear, and the edges of the cavities lose their distinctness, to regain it, however, when the "*osteoplaste*" (lacuna, corpuscle of Purkinje) shall have reached its ultimate dimensions by encroachment, as above stated, or by spontaneous scission. If the cartilage should have been vascular, the osseous *tissue* is now already formed—if it have been otherwise, the new osseous substance is perforated by *resorption* by numerous vascular canals, and by a similar process, canaliculi proceed from the "osteoplastes," and establish a general and perfect communication throughout the bone.

Let us now apply this to the teeth. "The first traces of the cement," says Koelliker, page 110: "which, consequently, are not formed by ossification of the tooth sacculus itself, I saw in a new born child, in the form of isolated fragments of an elongated or rounded figure, firmly engrafted upon the dentine of the yet short root, and which presented precisely the same appearance as formative osseous substance of the cranial bones. The smallest presented distinct lacunæ, * * * and upon the edges passed insensibly into a clear, cell-bearing blastema. * * * Now proportionate to the prolongation of the root such new spiculæ continually appear, and agglomerate gradually from above downwards into a single layer, upon which, then, from without, and in the same manner, there is again applied as much as is necessary to generate the entire thickness of the cement."

Here, at least, we have no difficulty in recognizing the process of "invasion:" and if a Haversian vessel should ever penetrate into the mass, as Tomes* has figured, and as we have seen in a preparation of our own, it occurs just as in any other bony tissue. Let us now turn to the dentine with regard to the "ossification" of which our author gives us valuable information, although "its formation out of the dentinal cells, is the obscurest point of the whole osteogenesis." To carry the reader with us unfatigued, we give a schema of all the parts, as they are described in Koelliker, concerned in the production of dentine and enamel.

| | | | |
|---|---|---|---|
| TOOTH SAC. | Enamel organ. | TOOTH SAC.<br>*Vascular Layer.*<br>Spongy body—a store-house of material.<br>Enamel membrane—a true epithelium. | TOOTH SAC. |
| | Tooth germ. | *Præformative Membrane.*<br>Dentine membrane—a stratum of cells.<br>Vascular parenchyma.<br>TOOTH SAC. | |

The *præformative membrane*, "corresponding to one of the superficial layers of the mucous membrane," covers the papilla of the future tooth; but although entering into most intimate relation with cement and ivory, is, as has been already stated, quite unimportant in the formation of the tooth. The "vascular parenchyma" of the germ has no specific office; but the "*dentine membrane*," a "cellular expansion," particularly attracts our attention. It rests *immediately* upon the vascular pulp, and consists of "cells," like those of cylinder epithelium, which, when detached from the inner side of a thin crust or cap of newly formed dentine, "are found nearly always to possess several nuclei; and, what most surprised me, are prolonged almost constantly at their extremity, which is turned *towards* the ivory, (or from the pulp,) into a shorter or longer filament, and

* J. Tomes, loc. cit. page 59.

this appeared to be either a simple shoot, or, what was more common, as if proceeding from their interior. The diameter of these filaments was, in the middle, 0.001 line, (the diameter of the canaliculi being 0.0006 to 0.0008 line K.,) their course straight or faintly undulating; their appearance homogeneous or clearer in the interior, like dentinal tubuli treated with muriatic acid; here and there they were forked, or gave out a delicate prolongation, or finally, showed a dilatation. Upon the extremity of the cells directed *from* the ivory (towards the pulp;) such filaments appeared very rarely, but they also presented a forked division. If the tooth-shell be removed, so as to leave the cells attached to the pulp, the entire margin of the latter appears like felt, or occupied by fine hairs, which originate broader from the dentinal cells, inflect themselves variously, and much attenuated, proceed outwards."

These "*cells* are pale, finely granulated," contain nuclei, which multiply by scission, as do also the cells, occasionally, in the long direction, (p. 108,) but commonly become elongated and constricted in the middle, without, however, separating into independent parts, (p. 103.)

"As the 'ossification' progresses," continues Koelliker, "it is not inconceivable that one and the same cell may extend itself during the whole formation of the dentine, yet much rather do I believe it possible that the dentinal cells are, from time to time, replaced by others."

The preceding observations, although much at variance with those of Nasmyth and Owen, agree, as the author remarks, with the researches of Schwann upon the teeth of the hog, and have, besides, a credible air of nature. His conception of the behavior of the several elements, by and through which the process of eburnation is accomplished, although the expressions of it do not every where harmonize, strikes us as being singularly near the truth. It differs, moreover, unintentionally, no doubt, from the opinion entertained in his article "Bone," but this seems to us confirmatory of the exactness of the author's observations, and is in close correspondence with the views we have set forth in speaking of the cement. But we are impressed with the belief that the word *corpuscle* might, with propriety, be substituted for "*cell*," whereby a greater similitude would be established between the *nidus* of the growing dentine and that of bone, and in this we think we are supported by the description of Koelliker himself.

The process of eburnation is thus summed up, (p. 107.) * * "At any rate, the fundamental substance of the dentine arises out of the cylindrical cells investing the pulp, which are more or less prolonged, blended with one another, and ossified. * * The canaliculi are derived from the nuclei of these cells, or, what is for the moment more probable, are the remains of their cell-cavities, of which the borders become more consolidated, and they consequently correspond to the lacunæ of bone. The divisions of the canaliculi may be explained by assuming that either the cells, from time to time, are separated longitudinally, which I truly believe to have seen, or that a succeeding cell should run into two preceding ones. As for the ramifications, nothing remains but a secondary process of resorbtion."

We would presume to arrange the facts thus: 1st. An amorphous substance uniting the "dentinal cells." 2d. Dentinal nucleated *corpuscles* corresponding to the *cavities* of transitory cartilage. 3d. Substitution of dentinal molecules for those of the amorphous substance, encroachment upon the corpuscles, which assume the form of filaments, and *disappearance of the nuclei* previously unchanged in form, (p. 107.) At this moment the canaliculi arise *in*, and their ramifications *between* the elongated corpuscles, just as at a later period a Haversian canal makes its appearance exceptionally in man, but normally in fishes, the rabbit or the elephant.

It is almost needless for us to remark that, as the invasion of dentine follows the ever-regenerating dentine membrane from without inwards, the pulp gradually shrinking upon itself until the crown of the tooth is completed. But the manner of the "deposit of the earthy salts" requires a moment's consideration.

P. 108. "There happens, at least in man, in the recently formed, but still slightly hardened ivory of morphological character, a deposit of earthy salts in such a manner that the whole seems to be composed of isolated spheroids. These spheroids, of which Czermak also makes mention, are seen in the first rudiments of dentine as well as in the later stages, most advantageously upon the edge of the root of one of larger teeth, regarded from without. * * * One easily assures himself that these spheroids are seated in the most external layers of the dentine, so that the smallest are ever the most inferior and the farthest outwards, and that they are nothing else than ossified spheroidal particles of the dentine, and not bodies deposited in the interstitial spaces. Similar spheroids lie not in the lower part only of the dentine, but throughout it, in a thicker or thinner layer, in the youngest portions which border upon the germ, * * and it is easy to perceive that the canaliculi penetrate through them."

The explanation of this appearance is found at p. 105:

"As the first manifestation of dentine, the outermost border of the apex of the germ, the (præformative membrane and the extremity of the dentinal cells, inclusively,) is seen to become yellowish and more dense in a small circuit. This change strikes deeper, extends over entire cells, and these, at the same time, are farther developed, until there results a rudiment of 0.048–0.072 line, &c. While this rudimentary shell is becoming larger and thicker, canaliculi appear in the part first formed, or that turned towards the enamel, and the *substance between them* grows darker and firmer. * * *

P. 109. "If the tooth formation proceed normally, calcareous salts are afterwards deposited between the spheroids, so that the ivory becomes perfectly homogeneous and clearer; in opposite instances the spheroids remain in greater or lesser number, and preserve between them spaces which are nothing else than the before-mentioned interglobular spaces, in completely ossified dentine."

The description of the development of the enamel is very nearly in accordance with the views of most exact and original observers; and we introduce a few passages of the author simply to present to the reader a unity in the consideration of this part of the history of the tooth, premising, as the author confesses, (p. 102,) that the study of the relations of parts in enamel, under process of development, offers less difficulty than in the other dental tissues.

P. 101. "The enamel organ may be regarded as a modified mucous tissue. The enamel membrane is the epithelium, the enamel organ, with the sacculus, answers to the mucous membrane proper, wherein it is remarkable, that the vessels penetrate so little into it, * * * and the spongy tissue, of great importance in the formation of enamel, is, in my opinion, * * a storehouse of material for the growth of the enamel membrane."

The "cells" of the enamel membrane—"a true cylinder epithelium"—have about the same dimensions as the "dentinal cells," that is, from 0.012 by 0.002 line, and beneath this layer may be seen a stratum of more rounded "cells," the young, undeveloped elements of replacement.

P. 102. "The deposit of lime begins in the cells, advances outwardly (from the dentine) in them, until at last, they have become entirely enamel fibres, and goes onward, at the same time, in new cells, so that the enamel layer grows thicker and broader. Meanwhile, the enamel membrane does not disappear, * * * but must be replaced by a new formative mass. How this occurs, whether by the intrusion of new cells, or by the unceasing successive growth of the pre-existing ones, is not yet decided; only this much is certain, that the enamel fibres, at every period, constitute a *continuum*, and thus, consequently, if really different cells successively have entered into their formation, they must have coalesced with one another."

The author failed to discover the glandular tubes* of the enamel pulp or "spongy tissue," but, as seen in the Cat, supposes that they bear some resemblance to certain *plaques*, multinuclear plaques of Robin, which may have relation to cell development in the reticular spaces of the enamel organ.

The alternation of material in perfect teeth, their reparation after injury, and their pathology, are each briefly considered. For the accomplishment of the two first processes, a system of tubes, pervading the whole tooth, had been esteemed by most observers to be analogically indispensable; and, accordingly, the canaliculi of the dentine, the osteoplastes and their diverging rudicles in the cement, and the irregular lacunæ† developed between them, left no doubt of the correspondence existing between these parts; and more recent authors‡ had described interprismatic canals in the enamel, thus rendering complete this association of the constituents of the tooth. Koelliker, however, denies the normal existence of these tubuli in the enamel, believing, that when such canals make their appearance, "they have probably quite another signification from those in

* See Todd and Bowman, loc. cit., p. 534.

† See Valentin, loc. cit., p. 730—Tomes, 1 c., p. 37.

‡ Todd and Bowman, loc., p. 531, &c., &c.

the dentine, to wit, that of excavations resulting from resorbtion"—(p. 117.) On the other hand, he admits, 1st, continuations of the canaliculi of the dentine; 2d, elongated cavities, dilatations of the same; and 3d, fissures in the middle and exterior parts of the enamel, but having no connection with the above—(p. 72.) We are inclined to regard these observations as insufficient, rather than incorrect, in proof whereof we adduce the instance cited by Valentin,* of "two back teeth of the horse, in which clefts, not only of the enamel, but also of the true substance of the tooth, and most probably the result of injury, were repaired with true osseous substance, as microscopic examination established."

Koelliker, however, speaks of "the perfect tooth, as not entirely deprived of alternation of material. What the lacunæ and their canaliculi are to bone, the canaliculi of the dentine, with their ramifications, the bone cavities and tubuli of the cementum, the spaces between the enamel prisms, are to the former. All these spaces conduct fluids during life, which are derived on the one hand from the vessels of the germ, on the other from those of the alveolar periosteum, and render possible a change of substance, although it be a slow one." Page 117.

In respect to pathology, not much is said, and this little bears no mark of originality. We find *Caries* spoken of as "not a simple dissolution of the salts by the fluids of the mouth, but a putrid decomposition of the organic part of the tooth, accompanied with development of infusoria and, fungi goes hand in hand with the former." (Page 120.) We would venture to suggest that the cryptoganic plants alone are sufficient to accomplish the disintegration of enamel and dentine, for placed in the most favorable circumstances, they may surely be supposed to effect as much as when attacking rock and castle they silently but certainly reduce them to dust. Well assured are we, however, that chemical action plays its part in this "doubtful" process; and we have seen, in pathological specimens in our possession, the canaliculi near a peripheral caries filled with the "brownish precipitate," but unlike the instances cited by Tomes, the tubuli were irregular and so much dilated that they

***G. Valentin, loc. cit., p. 731.**

exceeded in diameter their embouchures upon the walls of the cavity of the tooth.

The article is concluded with directions for the preparation of tooth sections adapted for microscopic observation, accompanied with indications concerning the conditions most favorable for the study of development—an addendum of great practical utility.

We cannot take leave of the author, without commending the zeal which has animated him in researches, and the honesty with which he has accorded full merit to other observers; yet, we could have wished that Art had lent its aid more effectually in the *illustrations,* since wood-cuts at best incompletely reproduce microscopic appearances.

*Baltimore, May* 15*th,* 1853.

